# The incidence and factors associated with anemia in elective surgical patients admitted to a surgical intensive care unit: a retrospective cohort study

**DOI:** 10.1186/s40001-024-01887-4

**Published:** 2024-05-19

**Authors:** Habtie Bantider Wubet, Lidya Haddis Mengistu, Negesse Zurbachew Gobezie, Begizew Yimenu Mekuriaw, Alemie Fentie Mebratie, Wosenyeleh Admasu Sahile

**Affiliations:** 1 Department of Anesthesia, College of Medicine and Health Sciences, Debre Tabor University, Debre Tabor, Ethiopia; 2 Department of Anesthesia, College of Medicine and Health Sciences, Addis Ababa University, Addis Ababa, Ethiopia; 3 Department of Midwifery, College of Health Science, Debre Tabor University, Debre Tabor, Ethiopia; 4 Department of Medical Laboratory Science, College of Medicine and Health Sciences, Debre Tabor University, Debre Tabor, Ethiopia

**Keywords:** Anemia, Incidence, Anesthesia, Surgery, Intensive care unit, Ethiopia

## Abstract

**Background:**

Anemia is a frequently reported and commonly documented issue in intensive care units. In surgical intensive care units, more than 90% of patients are found to be anemic. It is a hematologic factor that contributes to extended mechanical ventilation, sepsis, organ failure, longer hospitalizations in critical care units, and higher mortality. Thus, this study aimed to determine the incidence and identify factors associated with anemia in elective surgical patients admitted to the surgical intensive care unit.

**Methods:**

A retrospective follow-up study involving 422 hospitalized patients was carried out between December 2019 and December 2022 in the surgical intensive care unit after elective surgery at Tikur-Anbessa Specialized Hospital, Addis Ababa, Ethiopia. Data were gathered from the patients’ charts, and study participants were chosen using methods of systematic random sampling. SPSS 26 (the statistical software for social science, version 26) was used to analyze the data. Bivariable and multivariable binary logistic regression were used to examine associations between variables.

**Results:**

The incidence of anemia in elective surgical patients admitted to the intensive care unit was 69.9% (95% CI 65.4–74.5%). American Society of Anesthesiologists’ class III (ASA III) [AOR: 8.53, 95% CI 1.92–13.8], renal failure [AOR:2.53, 95% CI (1.91–5.81)], malignancy [AOR: 2.59, 95% CI (1.31–5.09)], thoracic surgery [AOR: 4.07, 95% CI (2.11–7.87)], urologic surgery [AOR: 6.22, 95% CI (2.80–13.80)], and neurosurgery [AOR: 4.51, 95% CI (2.53–8.03)] were significantly associated with anemia in surgical patients admitted to the intensive care unit.

**Conclusion:**

More than two-thirds of the intensive care unit-admitted surgical patients experienced anemia. An American Society of Anesthesiologists’ (ASA III score), renal failure, malignancy, thoracic surgery, urologic surgery, and neurosurgery were significantly associated with this condition. Early identification helps to institute preventive and therapeutic measures.

## Introduction

The World Health Organization (WHO) defines anemia as having a hemoglobin level of less than 13 g/dl (hematocrit 39) in males and less than 12 g/dl (hematocrit 36) in non-pregnant women. A reduced number of red blood cells results in a decreased ability of the blood to transfer oxygen to the tissues because hemoglobin is essential for carrying oxygen [1].

Anemia is highly prevalent and frequently observed in intensive care unit (ICU) -admitted patients after surgery. Approximately 67% (2/3) of patients admitted to the ICU on the day of admission have hemoglobin (Hb) less than 12 g/dl and 97% of patients in the ICU have protracted noticeable anemia [2]. In developing countries, the frequency of anemia is significant due to low socioeconomic status and inadequate healthcare services [3].

ICU anemia is strongly linked to poor surgical patient outcomes, including a higher risk of oxygen depletion (reduced blood oxygen-carrying capacity), blood transfusion, extended ICU stays, surgical site infections, resource consumption, prolonged need for mechanical ventilation, more complicated surgery, and advanced treatment [4, 5]. Furthermore, studies show increased requirements for re-intubation and weaning failure in those patients who are anemic [6].

Anemia in ICU-admitted patients poses challenges in both patient management and patient outcomes. This hematologic risk factor raises patient mortality and morbidity. Its adverse outcomes include congestive heart failure, respiratory failure, hypoxia, cardiac arrest, multi-organ failure, chronic kidney disease, failure of weaning from a mechanical ventilator, prolonged hospitalization, infection, and a greater chance of dying [7–9]. Anemia is also a great burden for anesthetists and anesthesiologists, as hemoglobin is one of the clinical parameters that determine anesthesia choice and service delivery to the patient [10].

Different techniques were tried to cope with patients with anemia as part of the management plan. Most of the time, anemia in ICU-admitted patients is managed by transfusing packed red blood cells, which improves oxygen delivery to the tissue and decreases tissue hypoxia [11].

However, the transfusion of blood to critically ill patients is potentially dangerous and carries a risk. As blood-transfused patients are immune-compromised most of the time, they are more likely to develop blood transfusion-related complications. Some of the reported complications following transfusion include ALI (acute lung injury), organ dysfunction, systemic infections, and death [12, 13]. Transfusion also increases hospital stay and resource consumption [14].

If anemia exists without obvious factors and if the patient is on certain medications, treatment can be done by withholding the drugs and putting the patient on corticosteroids [5].

However, anemia is an illness that can be treated and prevented and is frequently managed with fewer adverse effects without the need for blood transfusions if it is identified early to decrease blood transfusion and transfusion-related complications [15].

Even though anemia is highly prevalent in ICU-admitted patients, few studies have been conducted to investigate factors associated with it. Therefore, the purpose of this study was to ascertain the incidence of anemia and the factors associated with it in elective adult surgical patients admitted to ICU. It is advantageous for doctors and anesthetists to illustrate various anemia-related factors to lower the risk of transfusions, enhance clinical management, and enhance the quality of anesthesia and surgery.

## Methods

### Study design, period, and setting

A retrospective follow-up study involving 422 hospitalized patients was carried out between December 2019 and December 2022 in the surgical intensive care unit after elective surgery at Tikur-Anbessa Specialized Hospital (TASH), Addis Ababa, Ethiopia.

### Inclusion and exclusion criteria

#### Inclusion criteria

All patients 18 years of age and above who underwent elective surgery and were admitted to the surgical ICU have been included.

#### Exclusion criteria

- No records of baseline hemoglobin or hematocrit
- Hemolytic anemia as a direct cause of ICU admission
- Congenital causes of anemia such as sickle cell
- Patients on treatments for anemia were excluded from the study.

### Operational definitions

Hematocrit: the proportion of red blood cells in the blood. Healthy adults typically have hematocrits between 36 and 48% for women and 39–52% for men [16].

Hemoglobin: is a protein that contains iron in RBCs and is responsible for providing oxygen to the cells. It ranges from 12–16 for females and 13–17 for males [16].

Prolonged ICU stay: patients who are admitted to the ICU and stay for more than 8 days [17].

Post-surgery: the period starting from the immediate termination of surgery and till discharge [18].

Postsurgical anemia: is defined as anemia after surgery measured in terms of reduction of hemoglobin less than 13 g/dl for males and less than 12 g/dl for females according to the WHO [19].

Post-surgical anemia in the ICU: anemia developed during any period in the intensive care unit stay [20].

### Sample size determination

In the research area, the incidence of anemia among patients hospitalized in the surgical intensive care unit (ICU) and the associated risk factors were unknown. Using the single population formula, the sample size was determined with a 5% margin of error at a 95% confidence interval and a 50% incidence of anemia in surgical patients admitted to the intensive care unit:

$$n=\frac{\frac{za}{2}^{2}p({1}_{-p})}{{w}^{2}}$$

In this case, n = (1.96)^2^ 0.5 (1–0.5)/ (0.05)^2^ = 384, where *z*, 1.96; *p*,  0.5; *CI*, 95%; *w*, margin of error = 0.05.

To account for the non-response rate, we added 10% of the n (i.e., 384 + 38 = 422); as a result, 422 surgical patients who were hospitalized in the intensive care unit were included in the study.

### Sampling technique

The study participants were chosen using a systematic random sampling technique. The number of surgical patients admitted to the surgical ICU monthly was 40–45 patients. So, on average, 480 to 540 patients are admitted to the surgical ICU annually. Three years of data were taken retrospectively to establish the incidence of anemia and to search out the risk factors. Thus, in 3 years, 1530 patients were admitted to the surgical intensive care unit.

**Fig. 1:**
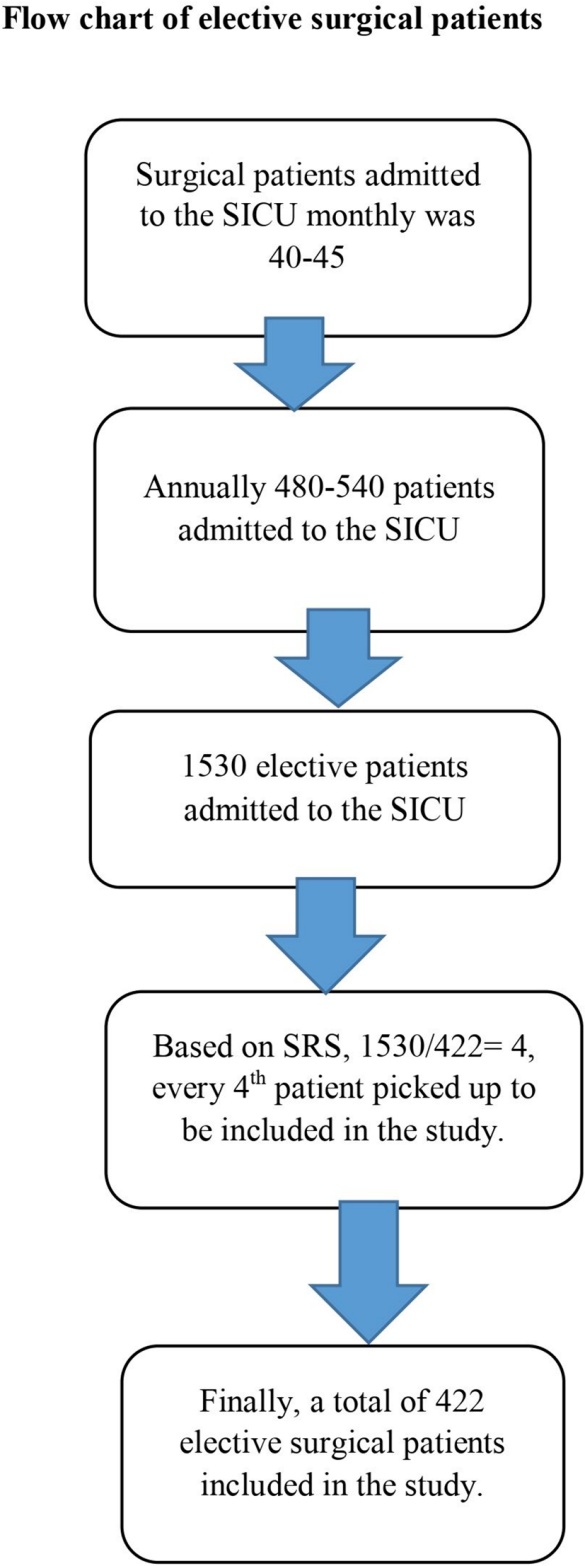
The flow chart of patients admitted to the surgical ICU after elective surgery

The sampling interval was based on systematic random sampling, and it was 1530/422 = 4. Therefore; every fourth patient was picked up to be included in the study (Fig. 1).

### Data collection procedure

Data has been collected from the patient’s chart by using a structured checklist. The questionnaire was designed to collect data on socio-demographic variables (age, sex, and residency), hematologic measurements (hemoglobin and hematocrit), previous surgery, medical status (hypertension, diabetes mellitus, renal failure, cancer, RVI, and heart failure), and drug history (Beta-blocker, calcium channel blocker, ACEIs, HAARTs, and chemotherapies). Blood loss, duration of surgery, duration on a mechanical ventilator, duration of ICU stays, and type of anesthesia employed were also investigated in patients’ medical charts.

### Data processing and analysis

Before being imported into Epidata version 4.6 and exported to SPSS version 26 for analysis, the data was first checked, coded, and entered. The collinearity diagnosis test (variance inflation factors, or VIF) and tolerance were used to screen for multicollinearity. The study employed binary logistic regression analysis to investigate potential relationships between the independent factors and the outcome variable. Variables in bivariable regression with a p-value less than 0.2 are added to multivariable logistic regression to assess the strength of the association. Lastly, variables are considered statistically significant if their p-value is less than 0.05. The model's fitness was evaluated using the Hosmer–Lemeshow goodness of fit test. The continuous variables were expressed using the mean and standard deviation. The categorical data have been expressed using percentages and continuous data using mean.

## Results

### Socio-demographic and clinical characteristics of study participants

This study was conducted on a total of 422 patients who were admitted to the ICU after elective surgery. Of the total study participants, 174 (41.2%) were men, 229 (54.3%) were from rural areas with a mean age of 48 (**± **17.7) years. The duration of surgery with mean ± standard deviation was 5.13 ± 1.25 h. The mean time of surgical patients waiting on mechanical ventilators and in the ICU was 8.0 ± 6 and 8.9 ± 6.6 days, respectively. The average volume of blood loss during the surgical procedure was 1380 (± 855) milliliters. Of those surgical patients, the majority of patients were ASA II (58.5%) (Table 1).

**Table 1:** Socio-demographic and clinical characteristics of ICU admitted patients following elective surgery

Variables	Category	Frequency	Percentage (%)
Sex	Male	174	41.2
	Female	248	58.8
Residency	Urban	193	45.7
	Rural	229	54.3
ASA	ASA I	75	17.8
	ASA II	247	58.5
	ASA III	100	23.7
Previous surgery	Yes	220	52.1
	No	202	47.9
Types of surgery			
Thoracic	Yes	93	22.0
	No	329	78.0
Abdominal	Yes	84	19.9
	No	338	80.1
Urologic	Yes	64	15.2
	No	358	84.8
Neurologic	Yes	123	29.2
	No	299	70.8
Vascular	Yes	20	4.7
	No	402	95.3
Head and Neck	Yes	44	10.4

ASA: American Society of Anesthesiologists, ml: milliliters, ICU: Intensive Care Unit

### Coexisting conditions and medical histories of patients admitted to the intensive care unit following elective surgery

Of the total study participants, 148 (35.1%), 90 (21.3%), and 84 (19.9%) study participants had histories of hypertension, cancer, and two or more diseases, respectively. Regarding treatment factors, 107 (25.4%), 102 (24.2%), 96 (22.7%), and 71 (16.8%) had histories of two or more medications, ACEIs, calcium channel blockers, and chemotherapies, respectively (Table 2).

**Table 2:** Clinical and medication history of elective surgical patients admitted to surgical ICU

Variables	Categories	Total (*N*)	Percent (%)
Hypertension	Yes	148	35.1
	No	274	64.9
Diabetes mellitus	Yes	34	8.0%
	No	388	91.9%
Renal failure	Yes	39	9.2%
	No	383	90.8%
Malignancy	Yes	90	21.3%
	No	332	78.7%
RVI	Yes	30	7.2
	No	392	92.8%
Heart failure	Yes	44	10.4%
	No	378	89.6%
Two and above disease	Yes	84	19.9%
	No	338	80.1%
Beta-blocker	Yes	32	7.6%
	No	390	92.4%
Calcium channel blocker	Yes	96	22.7%
	No	326	77.3%
ACE inhibitor	Yes	102	24.2%
	No	320	75.8%
Chemotherapy	Yes	71	16.8%
	No	351	83.2%
HAART	Yes	30	7.2%
	No	392	92.8%
More than two treatments	Yes	107	25.4%
	No	315	74.6%

### Incidence of anemia in surgical intensive care unit

**Fig. 2:**
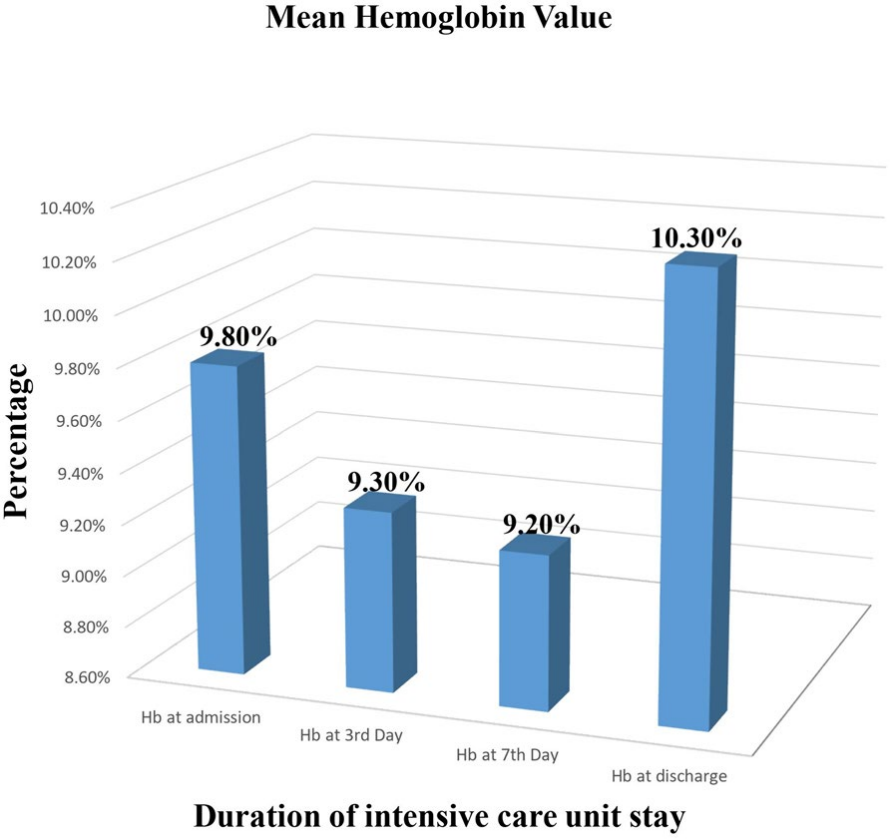
The mean hemoglobin value for patients admitted to the surgical ICU after elective surgery to the length of stay in the ICU

This study found that among patients hospitalized in the surgical intensive care unit following elective surgery, the overall incidence of anemia was 69.9% (95% CI 65.4% to 74.5%). The lowest and highest hemoglobin was noted on the seventh day of ICU admission (9.2 g/dl) and at the time of discharge (10.3 g/dl) respectively (Fig. 2).

### Factors associated with anemia in surgical ICU admitted patients

In a bi-variable logistic regression analysis, ASA class, renal failure, malignancy, heart failure, calcium channel blockers, ACE inhibitors, chemotherapy, thoracic surgery, urologic surgery, and neurologic surgery were discovered to be substantially associated with anemia (*p*-value < 0.2) and were candidates for multivariable logistic regression analysis. In multivariate logistic regression, ASA class III, renal failure, malignancy, thoracic surgery, urologic surgery, and neurologic surgery were found to have a significant association with the outcome variable (Table 3).

**Table 3:** Factors related to anemia in elective surgical patients admitted to the surgical ICU

Variables	Categories	Anemia		COR(95% CI)	AOR(95% CI)	*P*-Value
		Yes (*n* = 295)	No (*n* = 127)			
ASA status	ASA I	46 (10.9%)	29 (6.9%)	I	I	
	ASA II	166 (39.3%)	81 (19.2%)	1.29 (0.76–2.21)	1.47 (0.82–2.64)	0.2
	ASA III	83 (19.7%)	17 (4%)	3.08 (1.53–6.2)	8.53 (1.92–13.8)	0.005
Previous surgical history	Yes	161(38.1%)	59 (14.0%)	1.39 (0.91–2.10)	1.21 (0.75–1.95)	0.45
	No	134 (31.8%)	68 (16.1%)	I	I	
Renal failure	Yes	37 (8.8%)	2 (0.5%)	8.96 (2.13–13.8)	2.53(1.91–5.81)**	0.01
	No	258 (61.1%)	125 (29.6%)	I	I	
Cancer	Yes	77 (18.2%)	13 (3.1%)	3.09 (1.65–5.85)	2.59(1.31–5.09)**	0.008
	No	218 (51.7%)	114 (27.0%)	I	I	
Calcium channel blocker	Yes	80 (20.0%)	16 (3.8%)	2.58 (1.44–4.63)	2.23 (0.94–5.23)	0.09
	No	215 (50.9%)	111 (26.3%)	I	I	
ACEIs	Yes	83 (19.7%)	19 (4.5%)	2.23 (1.28–3.86)	1.48 (0.65–3.39)	0.4
	No	212 (50.2%)	108 (25.6%)	I	I	
Thoracic surgery	Yes	52 (12.3%)	41 (9.7%)	1.90 (1.07–3.39)	4.07(2.11–7.87)**	0.001
	No	243 (57.6%)	86 (20.4%)	I	I	
Urologic surgery	Yes	55 (13.1%)	9 (2.1%)	3.01 (1.44–6.29)	6.22(2.80–13.8)**	0.001
	No	240 (56.8%)	118 (28.0%)	I	I	
Neurologic surgery	Yes	99 (23.5%)	24 (5.7%)	2.17 (1.31–3.59)	4.51(2.53–8.03)**	0.001
	No	196 (46.4%)	103 (24.4%)	I	I	

^*^In multivariable binary logistic regression, statistically significant (p-value < 0.05)

COR Crude odds ratio (COR), AOR: adjusted odds ratio, CI: confidence interval, I: Reference

## Discussion

According to this study, the incidence of anemia in elective surgical ICU-admitted patients was 69.9% (95% CI 65.4% to 74.5%). The lowest hemoglobin level was noted on the seventh day of ICU admission (9.2 g/dl).

This study’s findings were consistent with those of other researches in Germany showing that 66.9% of ICU-admitted surgical patients had anemia (i.e. mean hemoglobin level less than 10 g/dl) during their ICU admission, and there was further decrement in mean hemoglobin level as the ICU stay increased [21].

Similarly, a Polish study found that nearly all patients became anemic during the first three days after ICU admission, and as many as 66% of patients were anemic on the day of admission [13]. Our study also showed that the first three days of ICU admission had a significant impact on hemoglobin concentration, with the biggest drops in hemoglobin occurring during this time and continuing to decline thereafter.

In contrast, the incidence of anemia at admission in our study was higher than the findings of the studies conducted in Greece (43.1%) [22], in Spain (63.31%) [4], in Western Europe (63%) [23], in the United States (57.8%) [24], and in Canada (61.3%) [25]. These differences might happen as a result of the fact differences in sample size and clinical characteristics of the study participants.

The incidence of anemia in our study was lower than in studies in India, reaching 97% within a week of ICU admission with hemoglobin or hematocrit levels below 12 g/dl (36%) [2]. This difference might have occurred because of differences in sample size and inclusion–exclusion criteria.

Further reports in Germany show the frequency of anemia in ICU-admitted patients was 98%, which is too high [26]. This variety has occurred because the study participants they included in this investigation were all on mechanical ventilators.

In Niger, the incidence of anemia in patients admitted to surgical ICU was 86.9% [27]. This was also high as compared with our result. This difference might be due to the inclusion of both emergency and elective surgical patients in their study.

According to this study, ASA physical status III, renal failure, cancer, thoracic, urologic, and neurologic surgery were found to be significantly associated with the incidence of anemia.

Compared to those patients with ASA I and II, patients with ASA status III had an 8.5 times increased risk of developing anemia. Other studies confirmed the ASA physical status class with preexisting co-morbidities increases adverse postoperative surgical outcomes [28]. As the physical status class of ASA increases, postoperative adverse events like morbidity and mortality increase too [28, 29]. As ASA class increases, coexisting medical conditions increase, the stage of disease may advance, and immunity declines resulting in decreased activity of reticuloendothelial cells which contributes to the development of anemia.

According to this study, the risk of developing anemia in surgical patients admitted to ICU was 2.5 times higher in patients with renal failure as compared with those patients without renal failure. This conclusion is consistent with research gained by other authors. Although anemia can occur at various stages of chronic kidney disease (CKD), the incidence of anemia and the severity of CKD are strongly correlated [30].

Renal hormones such as erythropoietin, which regulates the bone marrow's production of red blood cells, are thought to play a role in renal failure. As kidney disease progresses, other factors that lower red cell survival and inhibit marrow erythropoiesis may also contribute to anemia [30].

In our study, anemia was 2.6 times more likely to develop in patients having a history of malignancy than in patients who hadn’t a history of malignancy. Similar studies demonstrated that individuals admitted to the ICU with cancer had a higher frequency of anemia [31].

Compared to patients who were not having thoracic surgery, we found that patients who were having thoracic surgery were four times more likely to experience ICU anemia. Studies support the conclusions we have made. After undergoing pulmonary surgery, a retrospective examination of 465 patients in China revealed that 75.3% of patients in the intensive care unit had a postoperative anemia diagnosis [32]. This is also true in patients undergoing cardiac surgery in which the prevalence of anemia is high [33].

Patients who had undergone urologic surgery were 6.2 times more likely to develop anemia in the ICU than those who had not undergone urologic surgery. According to different studies, anemia is significantly associated with urologic surgery, and the magnitude is even higher in renal transplant surgeries [34, 35].

ICU anemia was frequently encountered in patients undergoing neurologic surgery, 4.5 times more frequently than in those who hadn’t undergone neurologic surgery. As evidenced by different published reports, anemia is a common scenario in patients undergoing neurologic surgery after ICU admission. This is supported by a study [36, 37].

The possible postulated mechanisms of anemia after surgery are described below. Anemia during surgery is a typical clinical scenario. Anemia that develops postoperatively resembles chronic illness anemia and is likely due to the impact that inflammatory mediators generated both before and after surgery have on the growth and survival of RBCs [38, 39].

## Strengths and limitations of the study

### Strengths

- Most of the studies on anemia were pre-operative and cross-sectional studies, so this study identifies the incidence and factors associated with anemia postoperatively after patients have been admitted to the surgical ICU using cohort studies.
- This study provides insights and clues about the incidence and associated factors of anemia in ICU-admitted elective surgical patients for future research; as such studies are limited in the area.

### Limitations

In this study, we used secondary data, and as a result, some important information regarding factors associated with anemia (e.g., nutritional status and living conditions) was not found during the chart review.

## Conclusion

Among surgical patients admitted to the ICU, the incidence of anemia is high. The majority of surgical intensive care unit admitted patients exhibit anemia during their course of stay. ASA physical status class III, renal failure, malignancy, thoracic surgery, urologic surgery, and neurologic surgery significantly increase the likelihood of anemia in surgical patients who are hospitalized in the intensive care unit (ICU). So, early screening and the institution of therapeutic measures are necessary to reduce the adverse effects of anemia. Preoperative screening of ASA physical status and medical conditions, early identifications and treatment of patients with comorbidities before surgery, and minimizing modifiable factors (i.e. duration of surgery and intraoperative blood loss) help to reduce the incidence of anemia in surgical patients admitted to ICU.

## Data Availability

If a legitimate request is made, the corresponding author will provide the data sets utilized and analyzed during the study.

## Abbreviations

ALI: Acute lung injury
ASA: American Society of Anesthesiologists
CBC: Complete blood count
DHS: Demographic Health Survey
Hb: Hemoglobin
HCT: Hematocrit
ICU: Intensive care unit
IRB: Institutional review board
PBM: Patient blood management
RBC: Red blood cell
SICU: Surgical intensive care unit
SPSS: Statistical Package for Social Sciences
SRS: Systematic random sampling
TASH: Tikur Anbessa Specialized Hospital
WHO: World Health Organization

## Declarations

### Conmpeting interests

There are no conflicting interests that the authors declare.
